# Ni(OH)_2_-decorated nitrogen doped MWCNT nanosheets as an efficient electrode for high performance supercapacitors

**DOI:** 10.1038/s41598-019-42281-z

**Published:** 2019-04-15

**Authors:** Sivalingam Ramesh, K. Karuppasamy, Hemraj M. Yadav, Jae-Joon Lee, Hyun-Seok Kim, Heung-Soo Kim, Joo-Hyung Kim

**Affiliations:** 1 Department of Mechanical, Robotics and Energy Engineering, Dongguk University –Seoul, Pil-dong, Jung-gu, 04620 Seoul, South Korea; 2 Division of Electronics and Electrical Engineering, Dongguk University –Seoul, Pil-dong, Jung-gu, 04620 Seoul, South Korea; 3 Department of Energy and Materials Engineering, Dongguk University –Seoul, Pil-dong, Jung-gu, 04620 Seoul, South Korea; 4 Department of Mechanical Engineering, Inha University, 100, Inha-ro, Nam-gu, Incheon, 22212 South Korea

## Abstract

In this study, nickel hydroxide nanoparticles (NPs) decorated with nitrogen doped multiwalled carbon nanotubes (N-MWCNT) hybrid composite was synthesized by thermal reduction process in the presence of cetyl ammonium bromide (CTAB) and urea. The as-synthesized Ni(OH)_2_@N-MWCNT hybrid composite was characterized by FTIR, Raman, XRD, BET, BJH and FE-TEM analyses. These prepared porous carbon hybrid composite materials possessed high specific surface area and sheet like morphology useful for active electrode materials. The maximum specific capacitance of Ni(OH)_2_@N-MWCNT hybrid nanocomposite in the two electrode system showed 350 Fg^−1^ at 0.5 A/g,energy density ~43.75 Wkg^−1^ and corresponds to power density 1500 W kg^−1^ with excellent capacity retention after 5000 cycles. The results suggest that the prepared two-dimensional hybrid composite is a promising electrode material for electrochemical energy storage applications.

## Introduction

Supercapacitors or ultra-capacitors are energy storage devices that have higher power density and better cyclic stability than conventional batteries. They are used as the most promising electrochemical energy storage devices with potential to eventually replace batteries for high-performance energy storage applications. Hybrid composites have been widely utilized in portable electronics, hybrid vehicles and backup energy systems for high-performance applications^1–3^. Based on energy storage mechanisms and properties, the supercapacitors can be classified into electric double layer (EDLCs) and pseudocapacitors^4–6^. In EDLCs, the electrodes generally consist of various forms of carbon, including activated carbons, carbon nanotubes, graphene and porous materials for supercapacitors^7–9^. Redox capacitors or pseudocapacitors employ transition metal oxides or conducting polymer as electrode materials.

In EDLCs, the energy storage mechanism involves electrostatic accumulation of charges at electrode-electrolyte interface between the electrode and ionic charges in the electrical double layer. However, in pseudo capacitors, stored energy is released in presence of reversible redox reactions or faradaic reactions occurring in electroactive materials. In addition, in EDLCs, capacitance is originated from a collection of charges at the electrode-electrolyte interface. To increase electrical conductivity and achieve high storage capacity, specific surface area and pore sizes are very important in EDLCs. Various graphene and carbon nanotubes structured composites have been widely used for electrochemical energy storage applications^7–9^.

The currently, recent developments of asymmetric supercapacitor is mainly focused on synthesis, properties, and performances of the state of the art materials for anode and cathodes in the supercapacitor applications. Mainly, the carbon-based materials such as porous carbon, activated carbon, carbon nanotubes, graphene, and graphene oxide materials are generally used for negative electrodes due to their high surface area and electrostatic charge-storage mechanisms at electrode/electrolyte interfaces. In particular, the pseudo capacitance of some metal oxides and nitrides have also been utilized as anode materials. The positive electrode containing the pseudocapacitive behaviours metal oxides and include carbonaceous materials. In addition, the electrode materials are design in the various nanostructured morphology of nanospheres, nanosheets, nanorods, nanowires, and nanoribbons. Recently, the studied two-dimensional materials such as transition metal dichalcogenides and transition-metal carbides for used in the current filed of supercapacitor applications^10^.

The asymmetric supercapacitors are an effective approach for extending the operating voltage window of the powder sources for supercapacitor and battery applications. These supercapacitor generally consists of a battery-type Faradic electrode (used as cathode) as an energy source and a capacitor-type electrode (anode) is a power source. Therefore, the asymmetric technology is expected to achieve an increased energy density and high cell voltage and improving the capacitance of cathode and anode materials have been widely studied. The carbon based materials are frequently used as anode in asymmetric supercapacitors. Because of the lower specific capacitance of carbon materials severely limits the energy density for supercapacitor for two electrodes. Metal oxide based anodes with nanostructures, such as MnO_2_, NiO, Co_3_O_4_, and Fe_2_O_3_, are promising electrode materials for asymmetric supercapacitors because of their high specific capacitance, two or three times higher than that of carbon/graphite-based materials^11^.

Based on these asymmetric electrode properties, the carbon nanotubes (CNTs) and reduced graphene oxide materials have also attracted significant attention in the field of biosensor, supercapacitor and electro catalytic applications due to their inherent potential properties of chemical stability, conductivity, flexibility, excellent catalytic activity and fast electron transfer reactions^12–15^. To improve the conducting property and surface area of MWCNT, nitrogen (N) was doped into the carbon matrix of MWCNT in the present investigation. Such N-doped process is very important for new generation of metal-free catalysts for electrochemical reactions. Nitrogen valence electrons are incorporated to the graphitic plane while π-electrons are generated in the carbon surface^16^. This may be due to π-electrons generation together with significant difference in electronegativity between nitrogen and carbon atoms. Therefore, the carbon-based materials contains unique properties such as high surface energy and polarization effect. N-doping can also improve both reactivity and electro catalytic performance of carbon materials. Therefore, *in-situ* doping involves direct incorporation of N atoms into carbon materials during the synthesis process in the presence of suitable catalyst.

Nickel oxide (NiO) is an important transition metal oxide that has attracted great interest due to its extensive applications in fields of catalysis, sensors and renewable energy sources^14–16^. A plenty of studies have been investigated the active electrode materials used like CuO, NiO, MnO_2_, Co(OH)_2_ and Ni(OH)_2_ for supercapacitors^16–22^. Amongst, nickel hydroxide, Ni(OH)_2_ materials have paid great attention due to their widely potential applications in alkaline rechargeable batteries, portable electronics and electric vehicles^2^. Further, it contains α and β phases. The crystalline β-Ni(OH)_2_ electrodes have been widely used for electrochemical applications due to their higher stacking density and stability when compared to α-Ni(OH)_2_. Numerous electrochemical studies have been reported the Ni(OH)_2_ electrodes on various carbon materials shows an improved electrochemical performance with excellent cyclic stability^23–26^. Electrochemical properties of Ni(OH)_2_/CNTs hybrid composite could be improved the specific capacitance and cyclic stability in presence of strong electrolyte like KOH at different concentrations. The electrochemical properties of Ni(OH)_2_/CNTs composite shows various specific capacitances and excellent stability of electroactive carbon materials have been shown^27^. Additionally, Ni (OH)_2_/MWCNTs hybrid nanocomposite can provide one-dimensional (1D) nanostructure owing to its high specific capacitance and excellent cycling stability and unusual electrical conducting properties.

Based on previous studies^28,29^, hybrid nanocomposites with different morphology based electrode materials have been synthesized for high-performance supercapacitors with excellent cyclic stability. In the present study, nanostructured nickel hydroxide oxide decorated N-MWCNT hybrid composite electrodes were synthesized and characterized for utilization in high performance supercapacitors for the first time. Systematic morphology and structural analyses revealed that the prepared hybrid electrode possessed high surface area with uniform pore size. Electrochemical properties of Ni(OH)_2_ @N-MWCNT hybrid composite (two and three) electrodes are displayed a high specific capacitance of two electrode is ~350 Fg^−1^ at 0.5 A/g and the detailed analyses are discussed below.

## Results and Discussion

### Structural results of the hybrid composite

**Figure 1:**
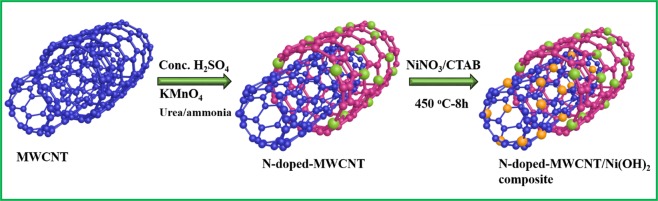
Schematic representation synthesis steps of Ni (OH)_2_ @N-MWCNT hybrid composite.

**Figure 2:**
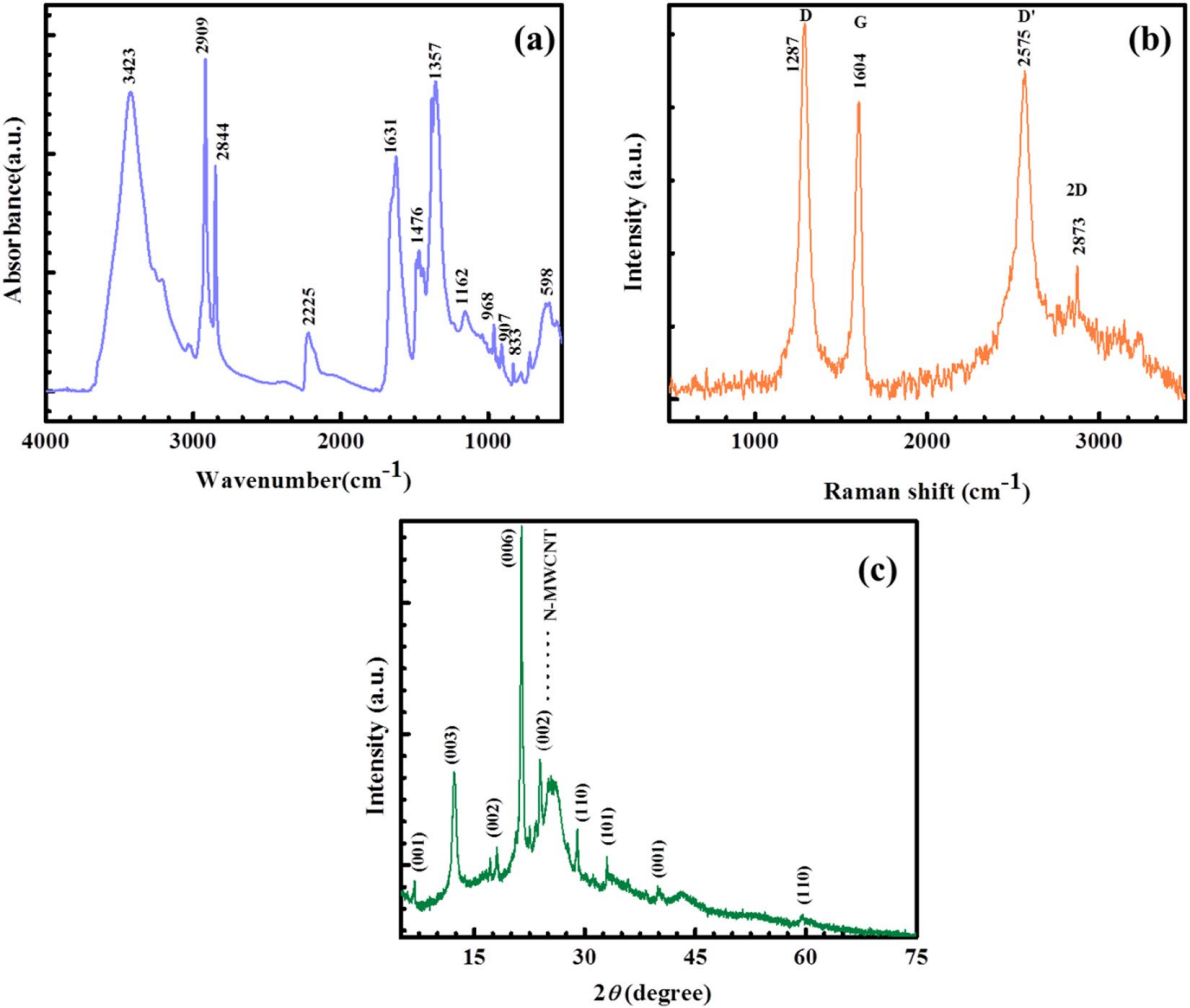
(**a**) FT-IR, (**b**) Raman and (**c**) XRD results of the hybrid composite.

The Schematic illustration of step by step synthesis of Ni(OH)_2_@N-MWCNT hybrid electrode is demonstrated in Fig. 1. The various structural interactions and complexation properties between N-MWCNT and Ni(OH)_2_ of the hybrid composite were analyzed through FTIR spectroscopy. The hybrid composite results are displayed in Fig. 2a. A broad band was observed at ~3495 cm^−1^. It could be represented to the stretching frequency of Ni(OH)_2_ present in the hydrogen bonded hydroxyl groups. The peaks appeared at 2844–2909 cm^−1^, indicating CH_2_ stretching mode from CTAB surfactant and other important functional modes such as C=O, C-H bending, C-O stretching, C-O-C stretching observed at 1631, 1476, 1357 and 1162 cm^−1^ respectively. There was a strong vibrational band in the region between 650 and 550 cm^−1^, especially at 598 cm^−1^ corresponding to Ni-O bond of Ni(OH)_2_. These results strongly suggest the presence of (NiOH)_2_ in the hybrid composite structure. The hybrid composite results also further confirmed that (NiOH)_2_ bonded to N-doped MWCNT surface in presence of CTAB surfactant and results compared to previous reports^28,30^.

Raman spectra of hybrid composite are shown in Fig. 2b. There were two prominent peaks at 1287 cm^−1^ and 1604 cm^−1^ represent that the D and G peaks from N-doped MWCNT, respectively. Generally, the G band indicates in-plane bond stretching motion of carbon (C, sp2) atoms of (E2g phonons). In addition, the peaks position of 2575 cm^−1^ (D’) and 2873 (2D) were also present in the hybrid nanostructure. The strong D band represents a large number of defects of N-doped MWCNT which is due to the reduction process of MWCNT. The intensity ratio of D and G peaks can be used to calculate disorder behaviours of carbon materials in sp2 domains^31^. The peak intensity of D and G are roughly calculated as (ID/IG = 1.08) represent that the large number of defects in the N-MWCNT surface. The peaks position at 3491 cm^−1^ and 2903 cm^−1^ are characteristic peaks of β-Ni(OH)_2._ These Raman spectral results confirmed that Ni(OH)_2_ was successfully anchored onto the surface of N-MWCNT.

Figure 2c represents XRD pattern of Ni(OH)_2_@N-MWCNT hybrid composite. Diffraction peaks at 6.94°, 12.34°, 17.10°, 18.08°, 21.04°, 24.03°, 25.82°, 29.12°, 31.11°, 33.10°, 35.92°, 40.0°, 51.42° and 59.63° corresponded to (001), (003), (002), (006), and (110) planes of Ni(OH)_2_@N-MWCNT surface confirmed by using standard JSPDS data (Card No. 14-0117).

All diffraction peaks showed hexagonal structure of Ni(OH)_2_. There was no impurity in the hybrid composite. Therefore, the XRD peaks proved that thermal reduction process played an important role in improving the crystalline nature, consistent with previous reports^31–33^.

**Figure 3:**
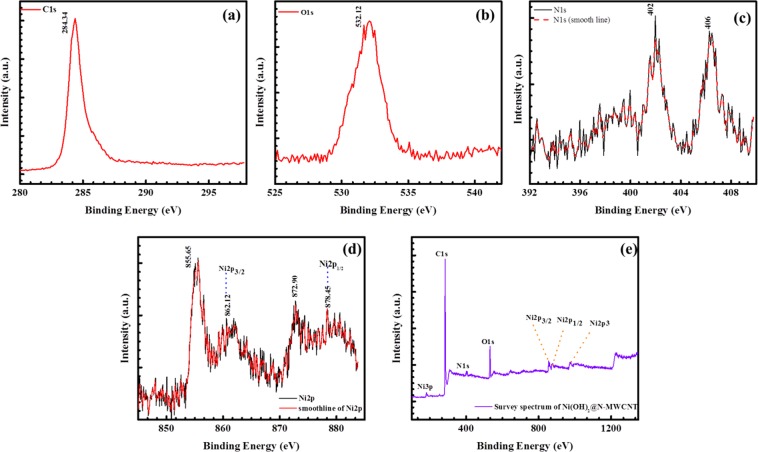
XPS results of (**a**) C1s, (**b**) O1s, (**c**) N1s, (**d**) Nip^3/2^ and Nip^1/2^ and (**e**) survey spectrum of the hybrid composite.

**Figure 4:**
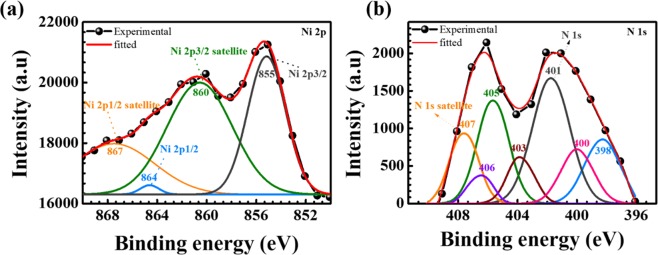
XPS fitting results of (**a**) Ni 2p and (**b**) N1s.

Surface chemical compositions of Ni(OH)_2_@N-MWCNT hybrid composite was analyzed by XPS. The XPS spectral results ranging from 0 to 1200 eV are shown in Fig. 3. Peaks at 855.65 and 872.90 eV indicates to Ni 2p^3/2^ and Ni 2p^1/2^ energy levels, respectively. Its corresponding fitting plot is represented in Fig. 4(a). Energy spin-energy separation of ~17.20 eV represented the characteristic of Ni(OH)_2_ phase and further confirmed with a previous report^32^. In addition, O1s peaks at 532.12 eV are associated with bound hydroxide groups (OH), carbon (284.34 eV) and nitrogen (402–406 eV) (Fig. 4(b)) present in the hybrid composite structure confirmed the bonding between Ni (OH)_2_ and n-doped MWCNT surface.

### Surface and morphological properties

**Figure 5:**
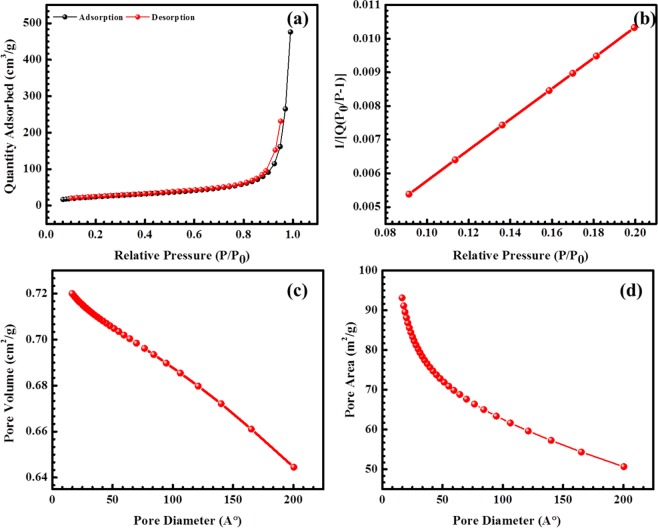
BET results of (**a**) adsorption-desorption, (**b**,**c**) pore volume and (**d**) pore area of the hybrid composite.

Figure 5 shows nitrogen adsorption/desorption isotherms of Ni(OH)_2_@N-MWCNT hybrid composite samples at 77 K and analyzed by mesoporous materials. BET surface area of the hybrid composite was ~250 m^2^ g^−1^, then that of pristine nickel hybrid oxides (~108 m^2^ g^−1^)^34,35^. Pore size distribution of the hybrid composite showed very thin microspores and mesoporous of 0.5 to ~2.5 nm and macrospores below the ~200 nm. The hybrid composite showed a wide pore size distribution with particle size of ~1.5–20 nm. These mesopores and macropores of hybrid composite can facilitate the migration of K^+^ and OH^−^ ions and enhanced the specific capacitance and cyclic stability in the electrochemical reactions^36^.

**Figure 6:**
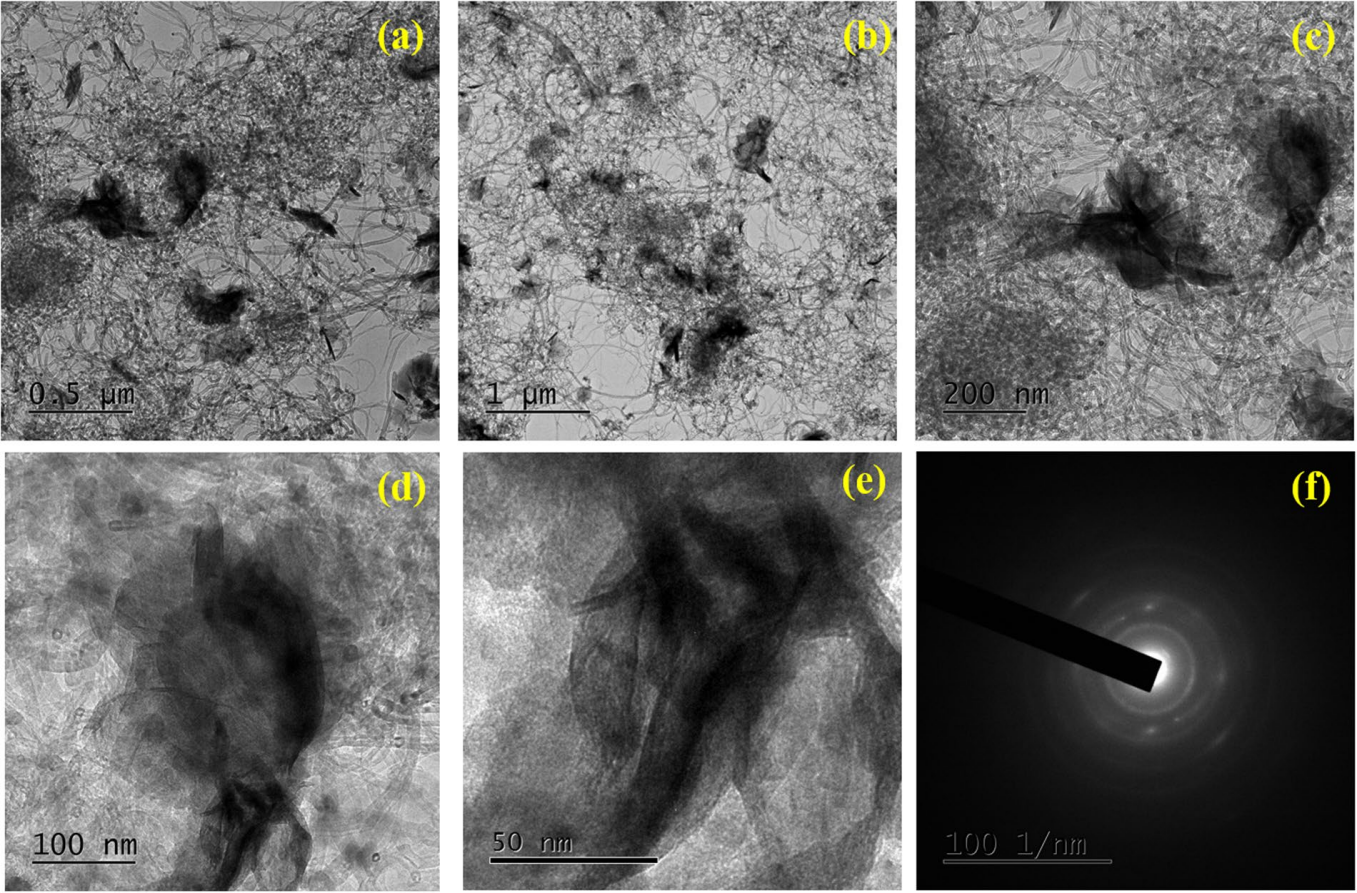
(**a**–**e**)HR-TEM images and (**f**) SAED pattern of the Ni (OH)_2_ @ N-MWCNT.

Morphology and structure of Ni (OH)_2_ @ N-MWCNT hybrid composite results were then studied by FE-TEM analysis. In these results (Fig. 6) of hybrid composite showed nanosheets like structure. The Ni(OH)_2_ nanoparticles are densely decorated over the surface of N-MWNT. In certain areas, aggregated nanoparticles are occurred, this may be due to mixing of N-MWCNT and Ni(OH)_2_. The SAED patterns corresponds to (003), (006), (110) and (002) patterns of Ni(OH)_2_ are observed in the hybrid composite and also consistent with previous reports^37^.

### Electrochemical properties of the hybrid composite

**Figure 7:**
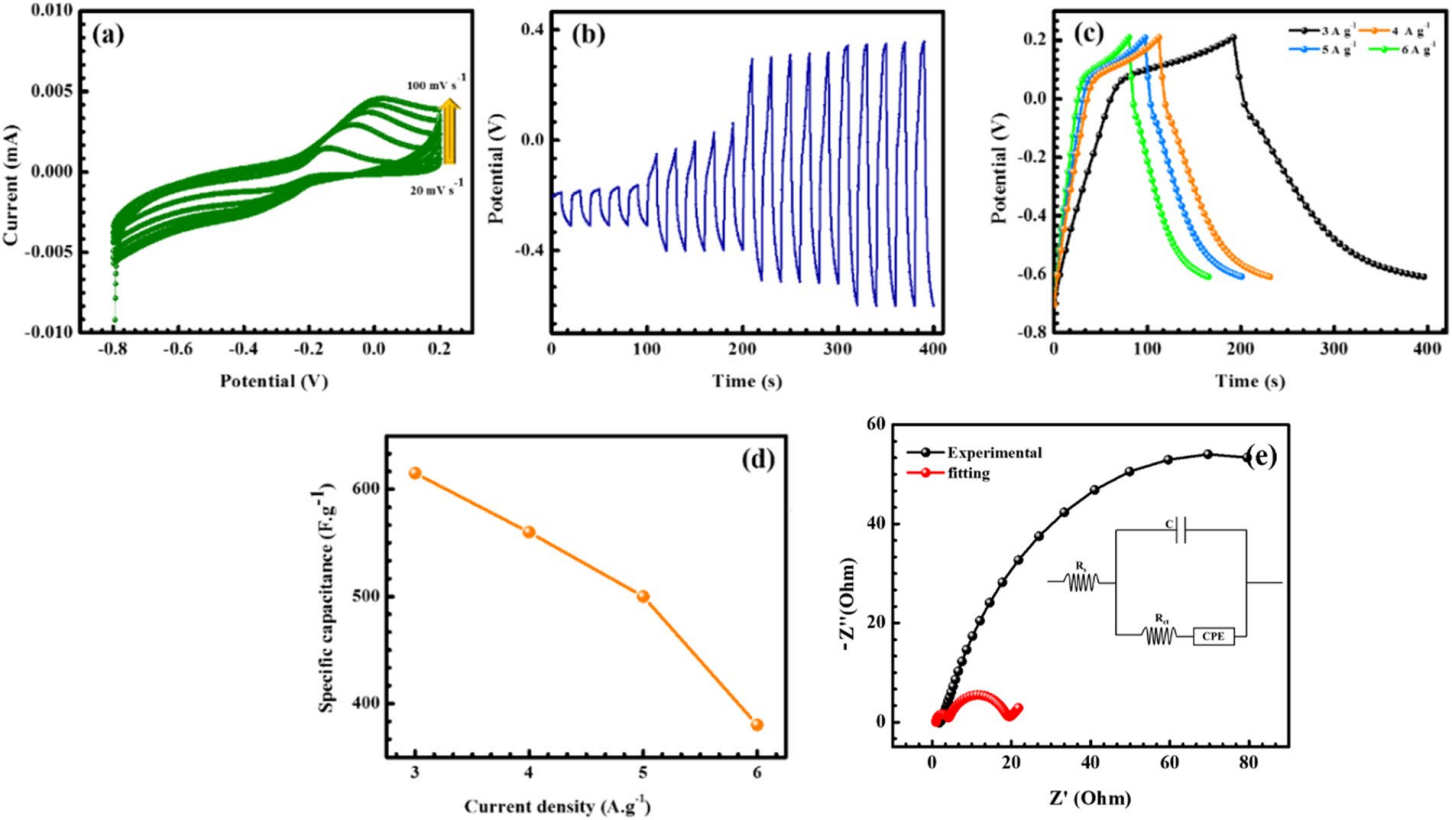
(**a**) CV at different scan rates (**b**) I vs t curve (**c**) GCD curve at different current densities (**d**) Capacitance at different current rates (**e**) EIS spectrum of Ni(OH)_2_ @N-MWCNT.

Electrochemical properties of pristine nickel oxide, nickel hydroxides, carbon materials and metal oxides for supercapacitor applications have been widely reported in previous studies^37–39^. The metal oxides and metal hydroxides previous reports very clearly showed that the specific capacitance and cyclic stability increases due to various surface morphologies present in the hybrid composite^38,40^. In the present study, electrochemical properties of Ni(OH)_2_@N-MWCMT hybrid composite was studied in the presence of 6 M KOH as electrolyte. Figure 7 shows CV profiles of Ni (OH)_2_ @N-MWCNT hybrid composite at different scan rates (20 to100 mV/s) in the range of potential −0.2 to 0.8 V. The shape of CV loops for the hybrid composite electrode was close to a rectangular shape, indicating double layer capacity behaviour. The electrode showed a higher current density response than MWCNT, indicating its good charge storage performance. These results indicate that the overall capacitance of the electrode is increased because of Ni(OH)_2_@N-MWCNT hybrid composite. Figure 7c shows results of galvanostatic charge–discharge tests of the electrode at different current densities form 3 to 6 A g^−1^. Therefore, the hybrid composite showed a linear triangular-shaped curve, indicating a double-layer capacitive behaviour. Figure 7c shows specific capacitance versus current density plot. The hybrid composite showed specific capacitances of ~615, 560, 500 and 380 F·g^−1^ at different current densities from 3 to 6 A g^−1^, respectively. The specific capacitance decreased when the applied current increased due to limited movement of electrolyte ions through electrodes. The composite exhibited higher capacitance (~615 F·g^−1^) than pristine MWNT due to decoration of nickel hydroxides onto the surface of N-MWNT which effectively prevented agglomeration of MWNT. This may be due to increasing the ion transport and improving the EDLC behaviour of the electrode materials. Moreover, the composite showed capacitance of 380 F·g^−1^ when increases to 6 A·g^−1^, indicating its excellent rate capability. The electrode showed only 10% capacitance loss after 5000 cycles at 3 A·g^−1^, indicating its outstanding long-time stability. This might be due to its strong interaction with nickel hydroxides and N-MWCNT’s rapid electron transfer and charge separation as well as its excellent conductive nature.

Typical Nyquist plots of Ni(OH)_2_ @N-MWCNT hybrid composite electrode are shown in Fig. 7f. The plot consisted of a semicircle at high frequency region (0.1 Hz to 100 Hz). The internal resistance of the hybrid composite electrode in an open circuit condition was evaluated. In the high-to-medium frequency region, one semicircle was related to faradic reactions and charge transfer in the electrochemical reaction^41–45^. The charge transfer resistance was found to be 3.2 Ω and 15 Ω respectively, before and after cycling process which demonstrating the stable electrochemical performance nature of the prepared electrode material. Consequently, the low ESR value (straight line) in the low-frequency region might be due to incorporation of hybrid composite which enhanced electrochemical properties of N-MWCNT surface^46–49^.

Cyclic stability is an important factor for electrode performance of supercapacitor in the electrochemical reaction via three-electrode system. As shown in Fig. 7b, the stability of hybrid composite as an electrode material was studied by repeating continuous galvanic-charge discharge (GCD) of 3 A g^−1^ for 5000 cycles. The GCD cycles of the hybrid composite electrode retained about ~90% of its initial specific capacitance after 5000 cycles, demonstrating its excellent cyclic stability. The high capacitance retention implies that the hybrid composite electrode is a suitable material for high performance energy storage devices.

**Figure 8:**
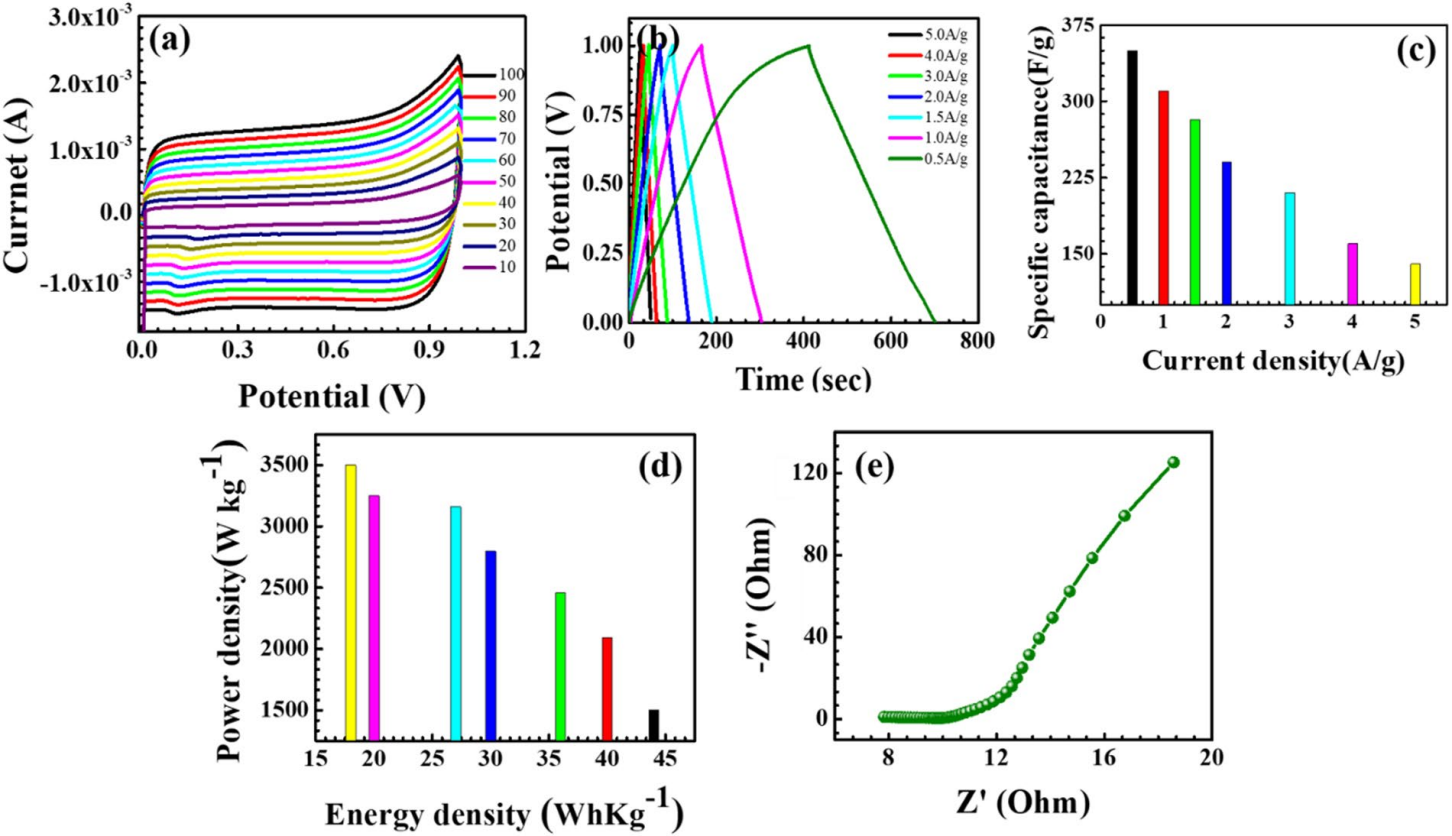
Asymmetric capacitor properties. (**a**) CV at different scan rates, (**b**) GCD curve at different current densities, (**c**) Capacitance at different current rates, (**d**) energy density vs. power density, (**e**) EIS spectrum of Ni(OH)_2_ @N-MWCNT.

In addition, the flexible solid state asymmetric supercapacitor device used to study the Ni(OH)_2_ @N-MWCNT hybrid composite electrode for practical applications. Ni(OH)_2_ @N-MWCNT hybrid composite asymmetric results are shown in Fig. 8(a–e). The CV curves in the potential window between 0 and 1.2 V at different scan rates are displayed in the Fig. 8(a). When the scan rates increase from 10 to 100 mV s^−1^, the shapes of CV curves are similar which indicating the good rate capability of the prepared electrodes. The electrochemical properties of the as-prepared ASC device was further evaluated by GCD tests at different current densities of 0.5 to 5 A/g, as shown in Fig. 8(b). The specific capacitance of the Ni(OH)_2_ @N-MWCNT hybrid composite ASC device can be calculated based on the loading mass of the active materials (Fig. 8(c)). The specific capacitances are found to be ~350, 315, 282, 240, 210, 160 and 140 F g^−1^ at the current densities of 0.5,1, 1.5, 2, 3, 4 and 5 A g^−1^ respectively, and retains 90% of its initial capacitance after 5000 cycles at a current of 0.5 A g^−1^. Figure 8(d) shows the Ragone plots that compares the power density and energy density^50,51^. It is quite observed from the Fig. 8(d) that the ASC shows the maximum energy density of ~43.75 Wh kg^−1^ with a power density of 1500 W kg^−1^ at 0.5 A g^−1^. Its relative nyquist impedance plot is provided in Fig. 8(e). In this asymmetric electrode device even at high current density of 5 A g^−1^, the energy density maintains at 17.50 Wh kg^−1^ at a power density of 3500 W kg^−1^ which suggests that the prepared composite electrodes could be utilized as a prime candidate in conventional electrochemical energy storage devices.

## Conclusions

In summary, Ni(OH)_2_@N-MWCNT hybrid composite was successfully synthesized *via* thermal reduction process. In this process, CTAB was an important surfactant to avoid agglomeration of nickel hydroxide particles. XRD diffraction peaks were proved to be hexagonal phase of β-Ni (OH)_2_. There was no impurity in the hybrid composite. BET surface area of the hybrid composite was ~250 m^2^ g^−1^, greater than pristine Ni(OH)_2_ sample (~108 m^2^ g^−1^). The hybrid composite showed wide pore size distribution with particle size of ~1.5–20 nm. The specific capacitance of the Ni(OH)_2_ @N-MWCNT hybrid composite showed the maximum specific capacitance of ~350 F g^−1^ at 0.5 A g^−1^ and retains 90% of initial capacitance after 5000 cycles. This can facilitate the migration of ions in presence of 6 M KOH electrolyte during the charge/discharge process. After 5000 cycles, only 10% of its initial capacitance was lost, demonstrating its good cyclic stability which in turn favors the way to utilize as potential candidate in high performance supercapacitors.

## Materials and Methods

All chemicals and reagents were used without any distillation process. Multiwalled carbon nanotubes (MWCNT-CMP-310F, Size 10–20 nm) were purchased from Lijiang Nanotech Co. Ltd, South Korea. Nickel nitrate Ni(NO_3_)_2_·6H_2_O, potassium permanganate (KmnO_4_), urea (Sigma-Aldrich, 98%), cetyltriemthyl ammonium bromide (CTAB), Sulfuric acid (H_2_SO_4_,), ammonium hydroxide (NH_4_OH), potassium hydroxide (KOH), polytetrafluorethylene (PTFE), Poly vinyl alcohol (PVA)MW = 77,000 were collected from Aldrich chemical company, South Korea.

### Synthesis of nitrogen doped N-MWCNT

Typical synthetic methods used to dope MWCNT materials with N include high-temperature arc-discharge process, CVD, solvothermal process (200–300 °C) and laser ablation methods were reported in detail^52–54^. Based on the thermal reduction chemical process of MWCNT synthesis as follows. MWCNT (3 g) was dispersed in DI water followed by well sonication for 2 h. The reaction solution was then added with 20 ml of ammonia and 1.2 g of urea followed by continuous stirring at 95 °C for 10 h. The resultant product of N-doped MWCNT was calcined at 350 °C for 8 h. Samples were then collected for further study.

### Synthesis of Ni (OH)_2_@N-MWCNT decorated hybrid composite

N-MWCNT (0.25 g) was taken in to 500 ml beaker and required 100 ml of distilled water then continuously stirred for 1 h at 95 °C. Stoichiometric amounts of Ni(NO_3_)_2_·6H_2_O (0.74 g), cetyl ammonium bromide (1 g) and urea (0.9 g) were then added into the MWCNT solution and stirred at 95 °C. Initially, the solution became turbid due to formation of Ni(OH)_2_ which was dissolved by addition of surfactant. Afterwards, the resulting transparent solution was kept under *vacuum* in a vacuum oven at 95 °C for 12 h. The resulting composite material was purified by ethanol followed by calcination at 450 °C for 8 h. Finally, hybrid composite samples were collected and stored in a desiccator to prevent moisture effect.

### Materials characterization

Ni(OH)_2_ @N-MWCNT hybrid composites were characterized by FTIR and RM200 confocal Raman spectro microscopy scanned in the range of 100 to 400 cm^−1^ in the presence of He and Ni laser beam source. XRD pattern of hybrid composite was studied using Rigaku Rotaflex (RU-200B) X-ray diffractometry in presence of Cu K_α_ radiation source. Morphology of the electrode was characterized by FE-TEM analysis using JEM-2010F (Hitachi S-4800, Japan). XPS analysis of hybrid composite was performed using XPS, ESCALAB 250Xi (Thermo Fisher Scientific, USA) in the presence of Al Kα radiation. Electrochemical properties of the hybrid composite were evaluated with CHI 760D (CH instrument Inc.). CV experiments were performed with the three-electrode system in the presence of 6 M KOH as strong electrolyte solution. In these experiments, the potential ranged from −0.8 to 0.2 SCE at a series of scan rates (20 to 100 mV/s). GCD curves occurred at various current densities (3, 4, 5 and 6 A/g) in the range of potential (−0.8 to 0.2). EIS analysis was characterized by using the experimental range of 0.1 Hz to 100 KHz and referring to open circuit potential. The electrochemical properties of the asymmetric cells were studied through cyclic voltammetry (CV), GCD, and EIS analyses via (VersaSTAT3 electrochemical workstation, Princeton, USA). The two-electrode system configured as counter electrode is connected together with reference electrode at same terminal.

### Electrode preparation

A conventional three-electrode system (CHI 760D, CH instrument Inc.) was used to characterize Ni(OH)_2_ @N-MWCNT hybrid composites in presence of 6 M KOH electrolyte solution. In this experiment, active material as a working, Pt wire utilize (counter) and reference electrodes (Ag/AgCl) for CV analysis. These working electrodes were fabricated by coating Ni wire electrode with 75 wt% active materials, 20 wt% conductive agent (carbon black) and 5 wt% polyvinylidene fluoride (PVDF) using N-methylpyrrolidone (NMP) as solvent. Electrochemical performance of the prepared hybrid composite was determined using cyclic voltammetry (CV) (20–100 mV/s), galvanostatic charge-discharge (GCD) (3 to 6 A g^−1^) and electrochemical impedance spectroscopy (EIS) measurements (0.1 Hz to 100 KHz). Results are discussed in the following sections.

The asymmetric device were fabricated as following methods: Ni(OH)_2_ @N-MWCNT hybrid composites electrodes were placed in the parallel and calculated 500 μm apart, then fixed with tape and inserted into a glass tube complete with 6 M KOH electrolyte. The KIMTECH paper was soaked in 6 M KOH solution and acted as the separator. The two electrodes were prepared by using a hybrid composite, conductive carbon black, and PTFE in the mass ratio of 85:10:5. The obtained slurry was then coated onto carbon fiber paper (with an active area of exactly 1 cm^2^) and dried at 90 °C for 30 min. The mass of hybrid composite electrodes E_1_ and E_2_ was 14 mg and 10 mg, respectively.
